# High‐Endurance STO:YSZ Optoelectronic Memristors with Vertically Aligned Nanocomposite Structure for Edge Detection

**DOI:** 10.1002/advs.202513646

**Published:** 2025-11-06

**Authors:** Jiacheng Wang, Jikang Xu, Xu Han, Weidong Sun, Fu Wang, Zhen Zhao, Kangbo Zhao, Yufei Shang, Biao Yang, Hong Wang, Haoning Liu, Xiaobing Yan

**Affiliations:** ^1^ Key Laboratory of Brain‐Like Neuromorphic Devices and Systems of Hebei Province, College of Electronic and Information Engineering Hebei University Baoding 071002 P. R. China

**Keywords:** edge detection, optical response, optoelectronic memristor, synaptic behavior, VAN structure

## Abstract

Edge detection plays a critical role in cutting‐edge domains such as real‐time monitoring and automatic driving, with optoelectronic device‐based real‐time image processing garnering significant attention. However, the poor endurance and unstable optical responsivity of conventional optoelectronic memristors constrain their application in highly integrated edge detection systems. In this study, a silicon‐based integrated optoelectronic memristor based on SrTiO_3_:(Y_2_O_3_:ZrO_2_) (STO:YSZ) vertically aligned nanocomposite (VAN) structure is introduced, where the conductive channels at the spatial vertical interface providing an effective transport pathway. The device achieves excellent endurance (10^8^ switching cycles) and stable multi‐band (405–650 nm) optical switching. Additionally, it also exhibits the ability of simulating biological synaptic plasticity, implementing optical image extraction and ASCII code transmission. Importantly, edge detection of real‐time road vehicle imagery is demonstrated via an optoelectronic memristor network. This work opens a promising paradigm for developing stable and high endurance machine vision systems based on optoelectronic memristor.

## Graphical abstract

A SrTiO_3_:(Y_2_O_3_:ZrO_2_) memristor featuring a vertically aligned nanocomposite (VAN) structure is developed. The vertically aligned conductive channels provide effective pathways for carriers, improving device performance, including high endurance (10^8^), and stable multi‐band optical switching. An edge detection system based on STO:YSZ memristors network is used to analyze road traffic condition images, opening new paradigms for building high performance biological visual systems.

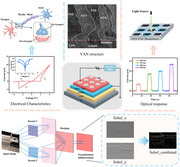

## 1 Introduction

As the requirement for artificial intelligence (AI) processing real‐time information increases, machine vision has become increasingly pivotal in AI applications like automated driving,^[^ ^1^ ^]^ real‐time surveillance,^[^ ^2^ ^]^ and automatic tracking.^[^ ^3^ ^]^ Machine vision enables machines to “see” and “understand” image and video information,^[^ ^4^ ^]^ with its operational efficacy being critically contingent upon the real‐time edge detection performance of the underlying hardware circuits. Edge detection serves as the foundation for AI decision‐making by extracting and simplifying image features. However, these machine vision systems are typically dependent on von Neuman computing architectures. Due to the separation of computation and storage, the systems must convert analog signals to the digital format and transmit them to the processor for computation.^[^ ^5^ ^]^ This inevitably leads to data redundancy, high energy consumption, and inefficiency in dense data processing and complex tasks,^[^ ^5^, ^6^, ^7^, ^8^ ^]^ making it difficult to meet the AI's requirements for real‐time data processing.

Recently, bio‐inspired brain‐like computing systems, based on optoelectronic memristors, have been reported. This is considered a powerful solution to break through the limitations of von Neuman computing architecture.^[^ ^9^, ^10^ ^]^ Optoelectronic memristors can directly sense light signals to generate responses, while also have the ability to temporarily memorize and sensory data and process visual information in real‐time.^[^ ^11^ ^]^ Currently, oxide materials are employed to fabricate optoelectronic memristors,^[^ ^12^, ^13^ ^]^ owing to their inherent optoelectronic coupling responsiveness.^[^ ^14^ ^]^ SrTiO_3_ (STO), characterized by a wide bandgap (≈3.2 eV) and a high dielectric constant (> 300).^[^ ^15^, ^16^ ^]^ Compared to traditional oxide materials (such as TiO_2_, ZnO), STO exhibits both excellent electron mobility and chemical durability,^[^ ^17^ ^]^ making it stand out among numerous oxides. Its photosensitivity originates from the acceleration of surface oxygen exchange by light irradiation, where photo‐oxygenation binding enhances the electrical conductivity of the STO membrane.^[^ ^18^, ^19^ ^]^ With their photosensitive properties and stability,^[^ ^17^ ^]^ STO materials have found extensive use in the development and application of memristors and photodetectors.^[^ ^20^, ^21^, ^22^ ^]^ However, pure STO‐based devices suffer from low conductivity and poor endurance,^[^ ^23^ ^]^ which greatly limit their application in edge detection system. Fortunately, vertically aligned nanocomposites (VANs) with interfacial coupling properties have emerged as key platforms for enhancing device performance,^[^ ^24^ ^]^ such as the introduction of thermochemically stabilized oxides into STO (e.g., MgO, Sm_2_O_3_, and CeO_2_).^[^ ^24^, ^25^, ^26^ ^]^ This structural incompatibility doped oxide structures tend to form spatially confined and periodically arranged conductive channels at the vertical interface between the nanopillar and the substrate. It can effectively avoid spurious conducting filaments and improve the stability and uniformity of the device.^[^ ^24^, ^27^, ^28^ ^]^ Notably, this unique vertically aligned structure provides an efficient transport pathway for photo‐generated charge carriers,^[^ ^29^, ^30^ ^]^ significantly enhancing their transport efficiency,^[^ ^31^ ^]^ thereby improving the device's optical response performance. On the other hand, Y_2_O_3_:ZrO_2_ (YSZ), characterized by high chemical stability and ionic conductivity,^[^ ^32^, ^33^ ^]^ demonstrates distinct advantages in nanocomposites with STO. The introduction of YSZ into STO promotes the ordered transport or oxygen ions in STO/YSZ composites, thereby enhancing the overall conductivity and endurance of the devices.^[^ ^34^ ^]^ Additionally, STO‐based optoelectronic memristors featuring VANs’ structure with high endurance and stable optical response can better meet the requirements of silicon‐based integrated edge detection systems. In this case, we aimed to grow VAN SrTiO_3_:(Y_2_O_3_:ZrO_2_) (STO:YSZ) films on Si by introducing YSZ. Compared to conventional memristors with lateral multilayer structures, the VAN's structure through the structural incompatibility between STO and YSZ contributes to the enhancement of endurance and overall performance in STO:YSZ devices. Thus, STO:YSZ nanocomposites are expected to serve as media with high endurance and stable photosensitive properties, paving the way for the use of VANs‐based optoelectronic memristors in edge detection.

In this study, we present a Pd/STO:YSZ/La_0.67_Sr_0.33_MnO_3_ (LSMO)/STO/P‐Si optoelectronic memristor with VAN structure, designed for edge detection of road vehicle conditions in autonomous driving. This highly oriented grown VAN STO:YSZ film results in well‐aligned conductive channels at the spatial vertical interface, enabling an effective transport pathway for carriers and contributing to enhancing the device's endurance and optical response. Notably, the device demonstrates robust non‐volatile switching behavior, high endurance (10^8^ switching cycles), and multi‐resistive states (retention up to 10^4^ s). Typical synaptic plasticity behaviors and advanced learning mechanisms including Paired‐Pulse Facilitation (PPF), Post‐Tonic Potentiation (PTP), Long‐Term Potentiation/Depression (LTP/LTD), Spike‐Amplitude‐Dependent Plasticity (SADP), and learning, forgetting, and re‐learning processes were simulated by continuously modulating the device's conductance. In a constructed convolutional neural network (CNN), the device achieves up to 91.5% accuracy in recognizing uppercase English letters from the Extended Modified National Institute of Standards and Technology (EMNIST) dataset. Additionally, it possesses stable optoelectronic characteristics at four wavelengths (405, 450, 520, and 650 nm), demonstrates stable optical switching in with/without light tests using laser sources with different parameters, and achieves an optical retention time of up to 10^4^ s. Especially, it achieves two functions of extraction of the optical image “H” and optoelectronic signal conversion of ASCII codes (F, Y, and H) in a simple communication system. Finally, we utilize the multi‐resistive state and optoelectronic properties of the STO:YSZ memristors to implement edge detection, which is ultimately realistic for image processing of real‐time road traffic conditions. This study demonstrates significant potential to advance the development of high endurance, programmable machine vision systems.

## 2 Results

### 2.1 Surface Morphology and Electrical Characterization

**Figure 1:**
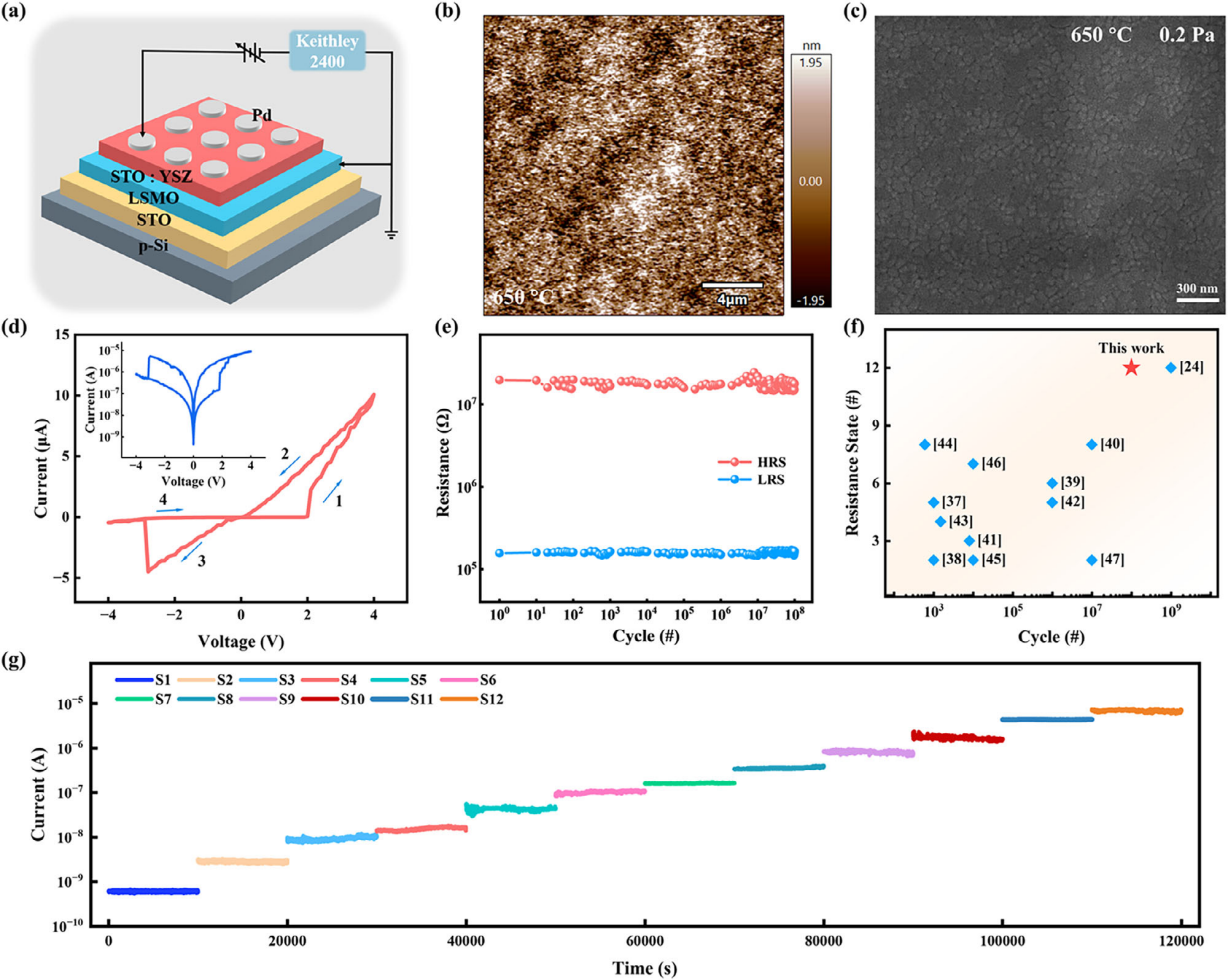
Characterization of Pd/STO:YSZ/LSMO/STO/P‐Si Memristor. a) Schematic illustration of the Pd/STO:YSZ/LSMO/STO/P‐Si device structure. b) AFM morphology of the device in 20 µm × 20 µm regions. c) SEM surface morphology of STO:YSZ devices at 45 k magnification and 300 nm scale. d) *I–V* characteristic of STO:YSZ memristors, with arrows denoting the voltage sweeping direction. The insert in the upper left corner shows the logarithmic form of the *I–V* curve. e) Endurance testing of the device, cycle endurance up to 10^8^. f) Endurance and multi‐resistance state comparisons of reported oxide‐based memristors, demonstrating the reliability and application potential of the STO:YSZ memristors. g) Stability testing of the devices across 12 distinct conductance levels, demonstrating stable multi‐level data storage capability.

**Figure** **1**a illustrates the electrical characteristics test schematic of an STO:YSZ memristor, featuring a Pd/STO:YSZ/LSMO/STO structure grown on a P‐Si (001) substrate, where STO is deposited directly as a buffer layer on the Si substrate, LSMO acts as the bottom electrode, STO:YSZ is the functional layer, and Pd serves as the top electrode. Figure S1 (Supporting Information) exhibits the X‐ray diffraction (XRD) test results for the device. In addition to the STO crystalline peak, the peak corresponding to the YSZ (002) crystalline phase is visible, indicating excellent crystallinity. To evaluate the quality of the STO:YSZ film, the surface morphology was characterized by Atomic Force Microscopy (AFM) and Scanning Electron Microscopy (SEM). The AFM image of the device film is depicted in Figure 1b, revealing a smooth and uniform morphology with a root‐mean‐square roughness (*Rq*) as low as 936.1 pm. Figure 1c shows the SEM image of the surface, further demonstrating that the STO:YSZ film possesses a homogeneous grain distribution, high density, and well‐defined crystalline structure. Additionally, homogeneous, nodular morphology facilitates enhanced synaptic properties of STO‐based devices.^[^ ^20^, ^35^ ^]^ Next, in order to investigate the resistive switching (RS) behavior of the STO:YSZ memristor, we tested the electrical properties of the device. With a direct current (DC) voltage applied to the top electrode and the bottom electrode grounded, the current–voltage (*I–V*) characteristics of the STO:YSZ memristor were tested by applying a sequential bipolar voltage sweep of 0 → +4 → 0 → −4 → 0 V, the *I–V* characteristics of the device as shown in Figure 1d. Figure S2 (Supporting Information) illustrates the resistance switching process of STO:YSZ memristors. The device is in its initial state when no voltage bias is applied. As the forward voltage increases, oxygen vacancies gradually accumulated. When exceeding the SET voltage (V_SET_), oxygen vacancy channels form, and device transitions from the high resistance state (HRS) to low resistance state (LRS). And as the reverse voltage exceeds the RESET voltage (V_RESET_), the device returns from LRS to HRS. The inset in the upper‐left of Figure 1d displays the logarithmic‐scale *I–V* characteristics, where a clear resistive behavior can be observed, and this voltage‐induced current modulation mechanism is crucial for modeling biological synaptic functions.^[^ ^28^ ^]^ Figure S3a (Supporting Information) exhibits the *I–V* characteristics of different devices, while the cumulative probability distribution of the SET/RESET voltage in Figure S3b (Supporting Information) demonstrates excellent consistency among devices. Additionally, the endurance and resistance state retention capability of the STO:YSZ memristor also directly determines the reliability and application, especially for the simulation of synaptic plasticity behavior.^[^ ^36^ ^]^ Endurance testing is employed to characterize the accuracy and reliability of the device, specifically to evaluate the conductance state switching across successive cycles under pulsed stimulation, while accounting for cycle‐to‐cycle and device‐to‐device variations.^[^ ^24^ ^]^ Figure 1e shows the endurance test results, where 126 data points were collected over the test cycle to characterize the reliability of the STO:YSZ device, with endurance cycles up to 10^8^. Significantly, we also compared the endurance and multi‐resistive states with other reported oxide‐based memristors,^[^ ^24^, ^37^, ^38^, ^39^, ^40^, ^41^, ^42^, ^43^, ^44^, ^45^, ^46^, ^47^ ^]^ as shown in Figure 1f. Compared to similar devices, STO:YSZ memristor demonstrate excellent endurance, which positions it as viable candidates for long‐term storage applications. Figure 1g shows the hold characteristics of the device across 12 different conductance states, each held for up to 10^4^ s without significant fluctuations, which provides the device with the possibility of performing high‐density memory computing. Crucially, the conductance of STO:YSZ memristor can be tuned by input signal, thus being essential for implementing high efficiency and accuracy image recognition and edge detection systems.

### 2.2 Microstructure Characterization

**Figure 2:**
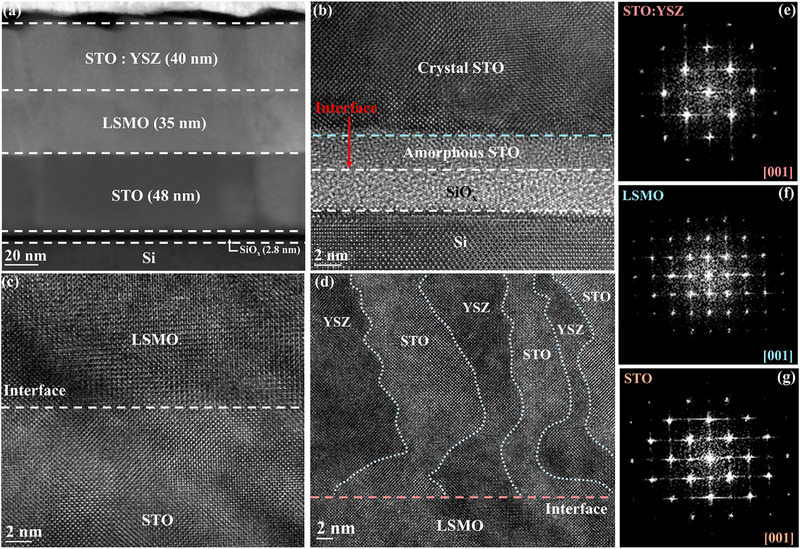
TEM images showcasing the layered structure of STO:YSZ/LSMO/STO/P‐Si stacks. a) Low‐magnification TEM image displaying the overall film structure, clearly exhibiting the thickness of the STO:YSZ, LSMO, and STO films. The high‐resolution TEM images of interfaces between b) the P─Si substrate and the STO, c) the STO and the LSMO, and d) the LSMO and the STO:YSZ, clear interfacial transition states and atomic arrangements can be observed. The FFT analysis of e) STO:YSZ, f) LSMO, and g) STO films, demonstrating excellent crystallinity of the devices.

To further analyze the microstructure of the devices, the STO:YSZ memristor films deposited on P─Si substrates were performed by transmission electron microscopy (TEM), where the main findings are shown in **Figure** **2**. Figure 2a shows a low‐resolution cross‐sectional image of the device, revealing the details of the STO:YSZ, LSMO, and STO layers (thickness of 40, 35, and 48 nm, respectively) grown on P─Si substrate. Figure 2b reveals a 2.8 nm silicon oxide layer formed through spontaneous oxidation of the P─Si (001) surface. This interfacial layer constitutes a primary contributing factor to amorphous phase formation in STO film. Figure S4 (Supporting Information) presents elemental analysis, confirming the expected stoichiometry throughout the STO:YSZ/LSMO/STO structure. Figure 2c,d shows high‐resolution images of the LSMO/STO and the STO:YSZ/LSMO interfaces, respectively, where the transitions at the interface can be clearly observed. Furthermore, due to the structural incompatibility and mismatched lattice constants between STO and YSZ,^[^ ^24^, ^27^, ^48^, ^49^ ^]^ the growth of the two components tends to extend along the direction of lower surface energy,^[^ ^50^ ^]^ thereby forming a unique vertically aligned structure. It is consistent with other reports.^[^ ^24^, ^27^, ^28^ ^]^ This VANs’ structure can induce the formation of regularly oriented and spatially confined conductive channels. Unlike the lateral diffusion of oxygen ions in heterogeneous structures, these channels allow rapid oxygen ion transport along the vertical nanocolumns in a direction that favors ionic mobility.^[^ ^25^, ^51^, ^52^ ^]^ This leads to a significant increase in the vertical ionic conductivity in VAN STO:YSZ film compared to normal STO film.^[^ ^53^, ^54^ ^]^ Meanwhile, the images indicate that the STO:YSZ/LSMO/STO structure grown on P─Si substrate exhibits high crystalline orientation, as confirmed by the fast Fourier transform (FFT) results from Figure 2e–g. The high‐quality growth of STO:YSZ memristors provides the physical foundation for the excellent electrical properties shown in Figure 1.

### 2.3 Simulation of Synaptic Behavior

**Figure 3:**
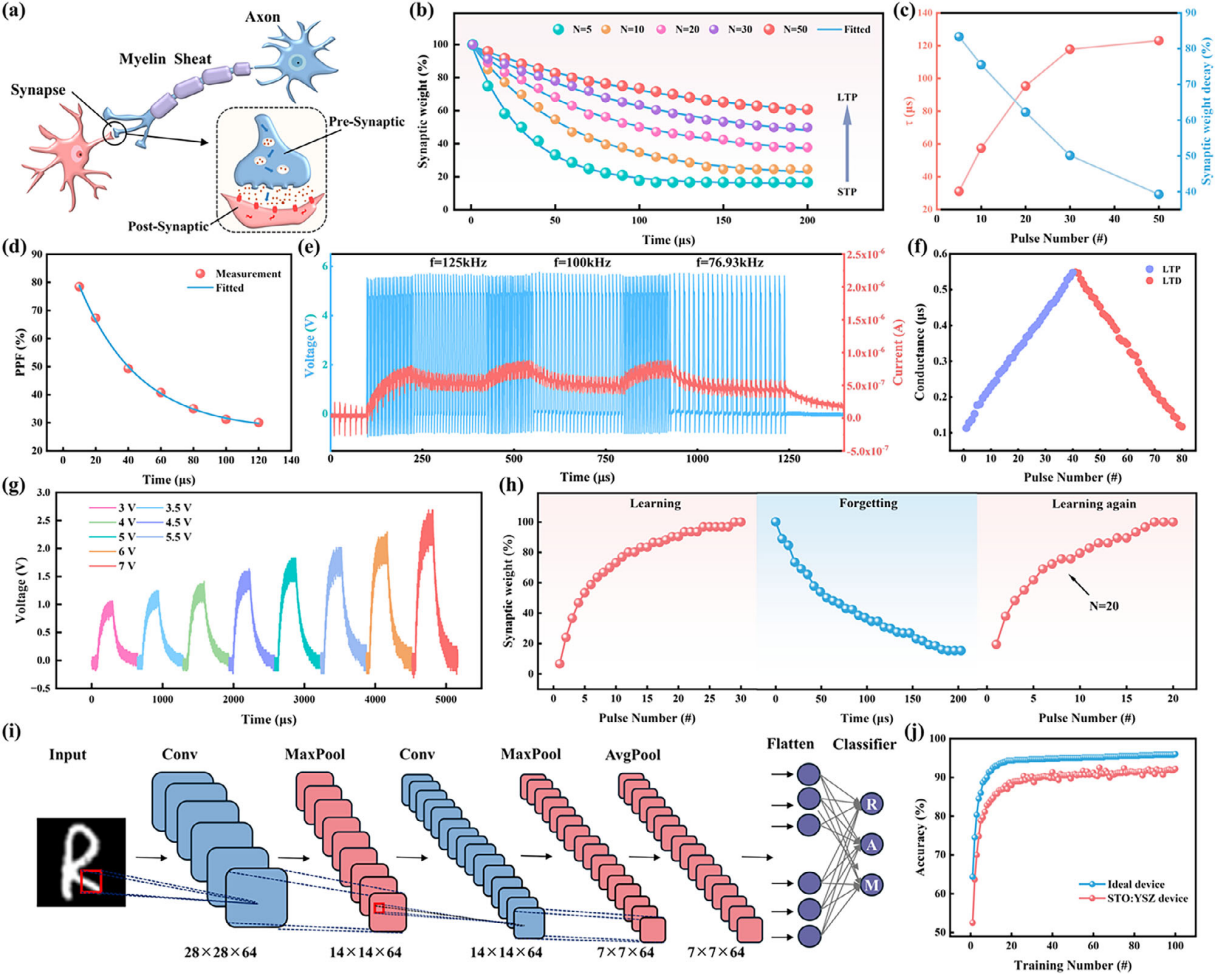
Characterization of artificial synaptic properties in STO:YSZ memristors. a) Schematic representation of neuronal and synaptic structures in the brain. b) Emulation of STP to LTP behavioral transition. c) Statistics on relaxation time and weight decay values in Figure 3b. d) PPF: pulse pairs of fixed amplitude 5 V and duration 20 us, with interval *Δt* increasing sequentially from 10 to 120 µs. e) Continuous PPF to PPD behavior transition. f) The LTP/LTD characteristics of the STO:YSZ memristors, with good linearity and symmetry. g) Simulation of the SADP mechanism. The duration and interval of the applied pulses are 5 µs, the number of pulses N is 25, and the amplitude are 3, 3.5, 4, 4.5, 5, 5.5, 6, and 7 V, respectively. h) The simulation of the behaviors of learning, forgetting, and re‐learning. Repeated learning takes less time to achieve previous memory level. i) Schematic of the convolution and pooling process in a STO:YSZ memristor‐based CNN for EMNIST handwritten letters recognition. j) Recognition accuracy of the handwritten letters R, A, and M for ideal device and STO:YSZ memristor.

The synapses fulfill a crucial role in the nervous system of the human brain, transmitting external stimulus signals from the presynaptic membrane to the postsynaptic membrane via neurotransmitters,^[^ ^55^ ^]^ thereby effecting the propagation of neural information between neurons, as shown in **Figure** **3**a. Synaptic weights are fundamental to the biological neural system, representing the magnitude of signals transmitted by synapses. During information transmission, changes in synaptic weights can enable organisms to respond to external stimuli and appropriately regulate their own behavior. The adaptive process of simulating synaptic weights facilitates the optimization of neural networks and improves the stability of the systems.^[^ ^56^ ^]^ To characterize the ability of the device to emulate biological synapses, multiple electrical pulse modulation tests were performed. Initially, electrical pulse signals with varying amplitudes and durations were applied to the 9 × 9 STO:YSZ memristors array to characterize the conductance modulation capability of the device. Bidirectional dimension of device conductance was observed, demonstrating the flexible conductance tunability of the STO:YSZ memristor, as shown in Figure S5a,b (Supporting Information). Figure S6a–c (Supporting Information) show the variations in the device's current response following the removal of applied electrical pulse signals with different amplitudes, intervals, and durations. When the stimulus disappears, the current level of the device doesn't drop abruptly but exhibits a gradual decay, closely resembling the biological phenomenon of the excitatory postsynaptic current (EPSC). Additionally, synapses serve as the core of the nervous system underlying learning, memory, and information processing. Their plasticity acts as a critical indicator of synaptic and neural system functionality. Synaptic plasticity is classified into short‐term plasticity (STP) and long‐term plasticity (LTP) according to the relaxation time (*τ*) following distinct stimuli.^[^ ^57^, ^58^ ^]^ Figure 3b shows the current variation of the STO:YSZ memristor in response to stimulation with varying numbers of pulse sequences, and a normalized fitting to the current decay trend was performed using the following equation:

(1) $$I_{t}=I_{0}+Aexp\left(-\frac{t}{\tau }\right)$$

where *I_t_* is denotes the current value at time t, *I_0_* represents the steady‐state current value, *A* is a prefactor, and τ is the relaxation time constant of the forgetting rate. As observed, with an increase in the number of pulses, the decay magnitude of synaptic weights decreases while the relaxation time prolongs over the same time period, thus simulated the transition of the STO:YSZ synaptic device from STP to LTP. Figure 3c presents the statistics on the relaxation times and decay magnitudes of synaptic weights. Figure S7a–c (Supporting Information) show the conductance modulation results of a single device, showcasing the STO:YSZ synaptic weights against pulse amplitude, duration, and interval. These findings highlight the influence of the parameters of the applied pulses on the conduction of the STO:YSZ memristors. This demonstrates the feasibility of precisely controlling the device conductance via pulse programming to emulate biological synaptic functions.

PPF is a form of STP, characterized by a significantly higher change in synaptic weights (Δ*W*) in response to the second pulse compared to the first during two consecutive stimulations. By fixing the amplitude of the applied pulse pair at 5 V, the duration at 20 µs, and varying the time interval (Δ*t*) between the two pulses in the pair, the Δ*W* of the STO:YSZ device can be tuned. Figure S8a (Supporting Information) shows the device's current response of following the application of the pulse pair. Figure 3d illustrates the relationship between the PPF index and the interval Δ*t*. The index is defined by equation:

(2) $$\mathrm{PPF}=\frac{\left(I_{2}-I_{1}\right)}{I_{1}}\times 100\%$$

where *I_2_* and *I_1_* denote the peak current responses of the STO:YSZ synapse to the second and the first pulses stimulations, respectively. Next, fitting the PPF index revealed a negative correlation between synaptic weights and the interval Δ*t*, with the fitting performed using equation:

(3) $$y=C_{1}e^{-\frac{t}{\tau_{1}}}+C_{2}e^{-\frac{t}{\tau_{2}}}$$

where *τ_1_* and *τ_2_* are the relaxation times, *C_1_* and *C_2_* are the initial fitting parameters. PTP is defined as the change in synaptic weights in response to the tenth pulse and the first pulse stimulation, and the final current response statistics at different Δ*t* are shown in Figure S8b (Supporting Information). It can be observed that the final current response decreases with increasing time interval of the pulse sequence, which is consistent with the updating pattern of biological synaptic weights. The relationship between the PTP index and the interval Δ*t* is similar to that of the PPF index, with statistics and fits as shown in Figure S8c (Supporting Information). The devices were then subjected to continuous pulse stimulation with a fixed amplitude and duration (5 V, 3 µs), and the frequency of the pulse sequence was varied at the end of the PPF test at 125, 100, and 76.93 kHz to observe the current response of the STO:YSZ device, as shown in Figure 3e. The current of the device gradually increases to saturation during the PPF interval, and the current response decreases after changing the frequency, corresponding to the transition from PPF to Paired‐Pulse Depression (PPD) behavior. Additionally, weight changes exhibit flexible and bidirectional plasticity, such LTP and LTD.^[^ ^59^ ^]^ It has been reported that highly linear and symmetric LTP/LTD properties enhance the accuracy of neural network,^[^ ^24^, ^60^ ^]^ making the study of bidirectionally tunable conductance in STO:YSZ memristors crucial. Figure 3f shows the LTP/LTD characteristics of the device achieved by applying a bidirectional pulse train with increasing and decreasing amplitudes, with overall good linearity and symmetry. The device conductance value increased from 0.113 to 0.547 µS under stimulation with 40 incremental pulse sequences from 3.125 to 8 V. Under stimulation with negative pulse sequences, the device conductance value decreased from 0.549 to 0.117 µS. Figure S9a (Supporting Information) shows the schematic of the tested pulses. Additionally, the 10 sets of LTP/LTD repeatability tests for the STO:YSZ memristors, as shown in Figure S9b (Supporting Information), demonstrate excellent linearity and consistency. The above results provide strong support for STO:YSZ memristors to model the complex synaptic mechanisms and cognitive functions of biological synapses.

Learning and memory are more advanced and vital cognitive functions of the human brain, enabling humans to adapt to nature and optimize their behavior. When neurons are subjected to multiple spike stimuli, synapses exhibit diverse plasticity effects, thereby enabling adaptation to the external environment. Simulating important cognitive and behavioral mechanisms in the human brain, such as Spike‐Rate‐Dependent Plasticity (SRDP), Spike‐Number‐Dependent Plasticity (SNDP), and SADP,^[^ ^61^ ^]^ facilitates the characterization of the ability of the device to modulate synaptic plasticity and weight levels. When the amplitude of the applied 25 consecutive pulses is increased, the response of the STO:YSZ memristor is proportional to the amplitude, as shown in Figure 3g. This is consistent with the SADP mechanism. Define the SADP index as the ratio of the maximum postsynaptic current (PSC) to the current response to the first pulse stimulation (I_25_/I_1_× 100%), and the index statistics are presented in Figure S10 (Supporting Information). Moreover, the synaptic weight of the devices exhibited strong dependence on rate and number of pulse sequences, corresponding to the SRDP and SNDP behaviors, as shown in Figures S11a and S12a (Supporting Information). Figures S11b and S12b (Supporting Information) present the statistical results of the SRDP and SNDP index. These represent the learning and adaptation processes of the human brain to varying degrees of external stimuli. These represent the learning and adaptation processes of the human brain to varying intensities of external stimulation. Figure 3h shows the simulation of the learning, forgetting, and re‐learning processes. Following the first learning and forgetting phases, the memory level of the initial learning was restored with significantly less relearning time. To further evaluate the performance of the devices, we simulated a CNN based on STO:YSZ memristors to recognize handwritten letters from the EMNIST dataset. Figure 3i illustrates the schematic of the convolution and pooling processes, and the whole network is divided into 3 parts (convolutional layer, pooling layer, and fully connected layer). A 28 × 28 grayscale image is input into the first convolutional layer, generating a 28 × 28 × 64 feature map. These maps undergo max‐pooling, reducing their size to 14 × 14 × 64. The second convolutional layer and max pooling then further downscale the feature map to 7 × 7 × 64. Finally, global pooling is performed to scale the image to the desired size, which is fed into the fully connected layer to recognize the letters R, A, M. The visualized feature maps of the letters after the first convolutional layer are depicted in Figure S13 (Supporting Information), with 64 feature maps gen for each other. Figure 3j shows the recognition accuracy of handwritten letters for the ideal device and the STO:YSZ memristor, with the ideal device achieving up to 96% accuracy. The STO:YSZ device achieves 91.5% recognition accuracy after 50 training epochs, which is close to the ideal device. These exceptional test results demonstrate potential of STO:YSZ devices for applications in high‐precision image processing systems.

### 2.4 Optoelectronic Characterization of STO:YSZ Memristor

**Figure 4:**
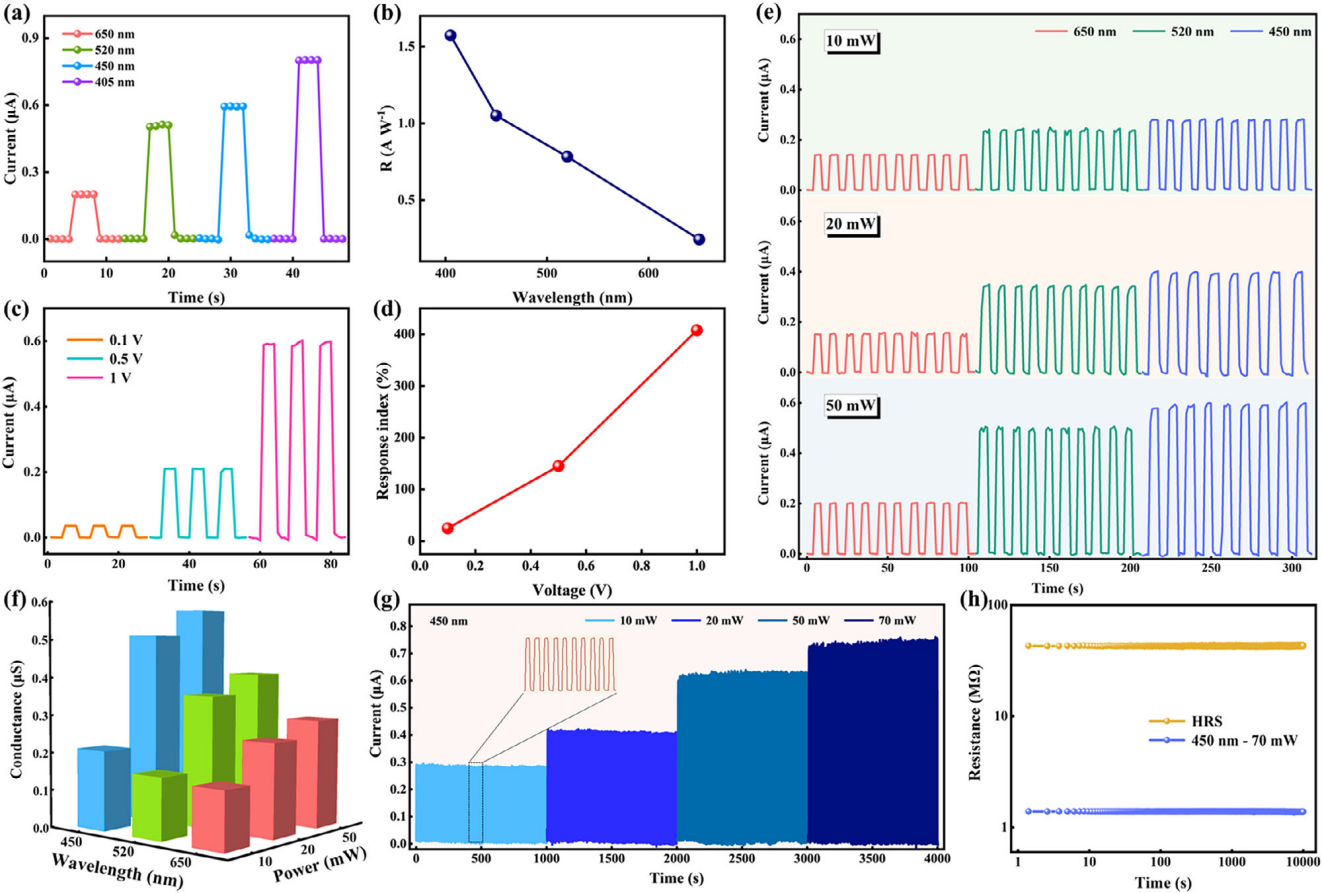
Optoelectronic characterization of STO:YSZ memristors. a) *I–T* characteristics of the STO:YSZ memristors at different wavelengths of light, with a fixed light source power of 50 mW and a 1 V reading voltage. b) The spectral responsivity (*R*) of the device to light stimuli of different wavelengths. c) *I–T* characteristics of the devices at different reading voltages, with a light source parameterized by a power of 50 mW and a wavelength of 450 nm. d) The optical response index of the device for different reading voltages. e) Current response of the device at different light power (10, 20, and 50 mW) and wavelengths (450, 520, and 650 nm). f) Conductance statistics of the device at different powers and wavelengths. g) Stability characteristics of the STO:YSZ memristor under optical stimulation at a wavelength of 450 nm, where 100 cycles of optical switching were performed at 10, 20, 50, and 70 mW, respectively. h) Retention stability of the devices under 450 nm, 70 mW continuous light stimulation.

Vision serves as a critical perceptual function in the human organism, where the retina transmits light signals to the brain for computational processing.^[^ ^62^, ^63^ ^]^ Optoelectronic devices process photosensitive characteristics and utilize light excitation in information storage.^[^ ^64^, ^65^ ^]^ Optical stimulation induces electron migration in the film, enhancing carrier concentration and influencing device conductivity. Surprisingly, the STO:YSZ optoelectronic memristors exhibit stable optoelectronic properties. Initially, the device's current response was characterized under varying light stimulation parameters. With a fixed light source power of 50 mW and a read voltage of 1 V, the current response of device measured at 405, 450, 520, and 650 nm light stimulation is presented in **Figure** **4**a. The device responds rapidly with an increase in current under external light stimulus. Following stimulus removal, its resistive state reverts to the initial condition, and the current response is negatively correlated with the wavelength. Figure 4b shows the device spectral responsivity (*R*) across different light wavelengths,^[^ ^62^ ^]^ with *R* calculated according to equation:

(4) $$R=\frac{I_{ph}}{P_{in}}=\frac{I_{light}-I_{dark}}{P_{in}S}$$

where *I_p_* denotes the photocurrent value, defined as the difference between the photocurrent (*I_light_*) and dark current (*I_dark_*), *P_in_* represents the incident light intensity on the devices, and *S* signifies the effective area. The *R* characterizes the response capability of the STO:YSZ memristors to varying light wavelengths.

Variations in read voltage also influence the current response of the device under light stimulation. Figure 4c demonstrates the variation of the device photocurrent response under different read voltages, with a fixed light source wavelength of 450 nm and power of 50 mW. Notably, the photocurrent response increases with rising read voltage, and the response index at three specific read voltages is shown in Figure 4d. The response index of the device under different optical parameters validates the accuracy of the findings of Figure 4a from a longitudinal perspective, as shown in Figure S14 (Supporting Information). Figure 4f shows the conductance statistics of the STO:YSZ memristor under different optical parameters, demonstrating that adjusting the light power and wavelength alters its conductance state. To further validate the stability of the device optical response, investigating its retention characteristics at with/without light is essential. Figure 4f and Figure S15a,c (Supporting Information) show the current–time (*I–T*) characteristics of the device during 100 optical switching cycles under optical stimuli of different power and wavelength, with a stable response during switching. The device's photocurrent response demonstrates a positive correlation with the light power. Additionally, the modulator effect of light illumination at different wavelengths on the device's LRS was investigated. Figure S16a (Supporting Information) vividly illustrates the distribution of photogenerated carriers within the STO:YSZ film. The photocurrent response is shown in Figure S16b (Supporting Information), the LRS current exhibits an increasing trend with wavelength decreases. These excellent optical responses can be attributed to the conductive channels influencing the formation of oxygen vacancy channels^[^ ^66^ ^]^ and the migration efficiency of photogenerated carriers,^[^ ^29^, ^30^ ^]^ thereby enhancing the optical response of the devices. The retention characteristics of the devices at with/without light were tested, as shown in Figure 4h and Figure S15b–d (Supporting Information). Under continuous light stimulation at 70 mW and 450 nm, the resistance value of the device in the open state is 1.4 × 10^6^ Ω (corresponding to the blue line in Figure 4h). The device demonstrates high stability in both states, with retention times of up to 10^4^ s.

**Figure 5:**
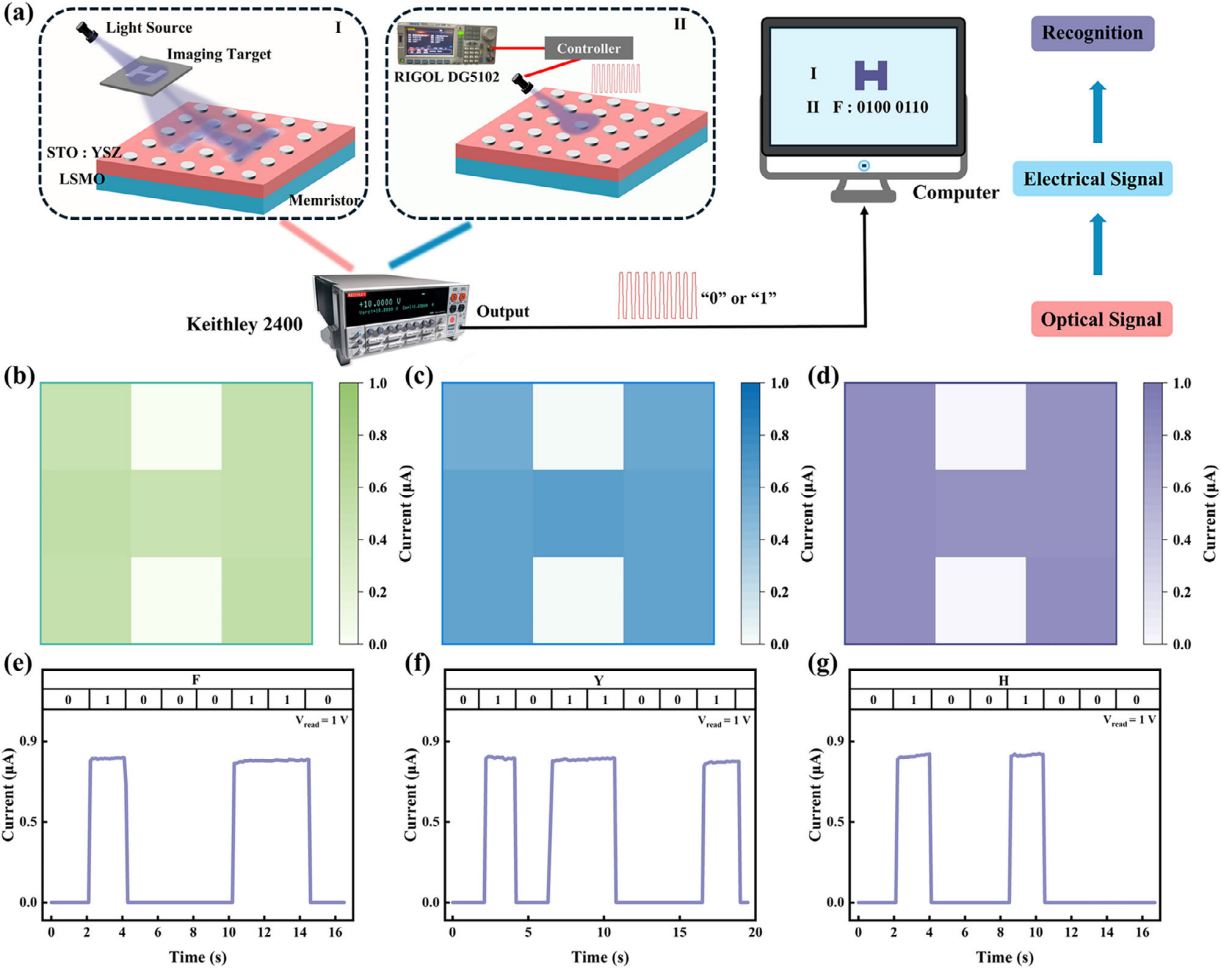
Optoelectronic transmission functions. a) Schematic illustration of a simple optoelectronic communication system based on STO:YSZ memristors. Ι: Image extraction from a 3 × 3 STO:YSZ device array, II: Optical‐to‐electrical signal conversion for ASCII code translation. Image extraction results of the 3 × 3 STO:YSZ optoelectronic memristor under light irradiation at wavelengths of b) 405 nm, c) 450 nm, and d) 520 nm, respectively. Transmission results of ASCII codes for letters e) F, f) Y, and g) H.

To validate the potential of STO:YSZ memristors for optical communication and machine vision applications, a simple communication system was developed to verify the device's optical sensing performance. The system consists of three components: optical signal input and reception, signal acquisition and transcoding, and data output, as shown in **Figure** **5**a. Response speed is a metric for evaluating photodetector performance, as it directly reflects the capability of the device to track rapidly switching optical signal.^[^ ^67^ ^]^ In this study, the response speed of the device was tested using 50 mW light source at wavelengths of 405, 450, and 520 nm, respectively, with results presented in Figure S17a–c (Supporting Information). The STO:YSZ memristors exhibit millisecond‐scale response speeds to optical stimuli of different wavelengths, a critical requirement for high‐speed and optical detection, imaging, and information synchronization systems. Subsequently, different wavelengths of light (405, 450, and 520 nm) were projected through the mask to illuminate the letter “H” onto a 3 × 3 array of STO:YSZ devices, with the read voltage set to 1 V. Image extraction was achieved by comparing the current response in light and dark conditions, with the array's schematic shown in Figure S18a (Supporting Information). Figure 5b–d illustrate the letter “H” extraction results by the array at different wavelengths, clearly showing that the image is extracted and its clarity improves as the wavelength decreases. Figure S18b–d (Supporting Information) shows the light‐dark current statistics of the devices within the designated region at varying wavelengths of light. The response values exhibit excellent stability, which concurrently corroborates the accuracy of the extraction results. In addition, the real‐time optoelectronic signal conversion capability of the STO:YSZ memristors was tested. The letter information is encoded into the oscilloscope in square wave. Subsequently, the oscilloscope transmits the control information to the controller, which directs the light source to emit optical signals (1: light source active, 0: light source inactive). The STO:YSZ memristor then converts the received optical signals into electrical signals via a digital source meter. Following that, the electrical signals are processed by a computer, decoding the ASCII code into corresponding letter output. The results of the ASCII code transmission by the STO:YSZ memristor for letters F, Y, and H are shown in Figure 5e–g. Thus, the STO:YSZ memristors exhibit excellent optoelectronic characteristics, making them highly promising for applications in optoelectronic communication and machine vision.

### 2.5 Edge Detection Implementation Using STO:YSZ Memristors

**Figure 6:**
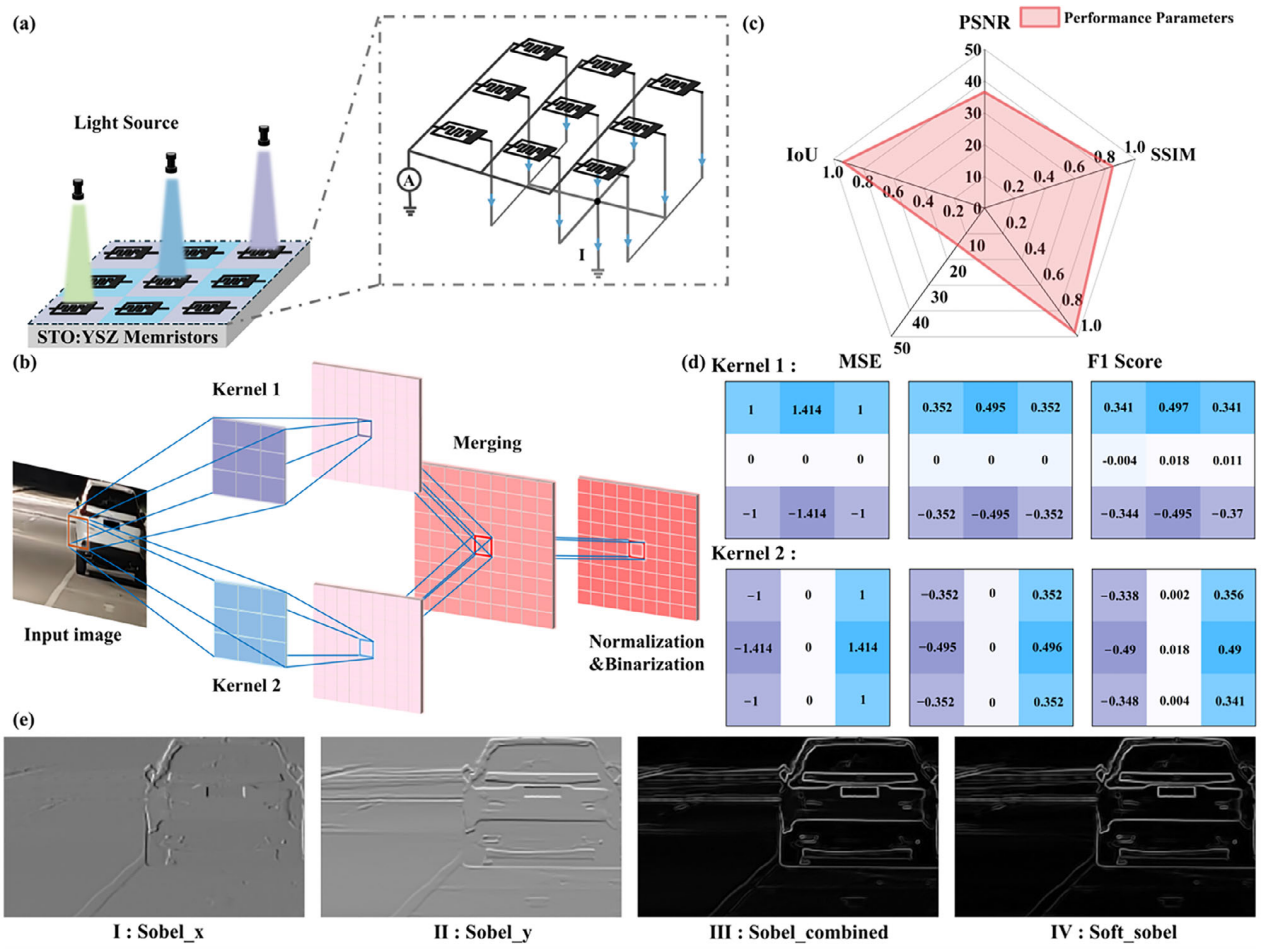
Edge detection based on STO:YSZ optoelectronic memristors. a) Schematic of a single‐layer photoreceptor convolutional kernel circuit structure, hardware‐implemented by a 1 × 9 (M = 1, N = 9) SRO:YSZ optoelectronic memristor network. b) Real‐time vehicle conditions on the road obtained by edge detection. c) Performance parameters from the comparison of edge detection results between the STO:YSZ optoelectronic memristor‐based system and software, including PSNR, SSIM, F 1Score, MSE, and IoU. d) From left to right: theoretical dimensionless weights, theoretical photoresponsivities scaled from dimensionless weights, and actual weights right after programming, for kernel 1 and 2. The unit of photoresponsivity is mA W^−1^ (not shown). e) Edge detection results for the road condition image. Ι: output image after convolution with kernel x, II: output image after convolution with kernel y, III: final edge detection's result for the road condition image, IV: software‐obtained edge detection results.

Edge detection, a fundamental function in image processing, plays a crucial role in machine vision. It can execute in situ multiply‐accumulate (MAC) operation between the optical response matrix and an input image.^[^ ^4^, ^11^ ^]^ The STO:YSZ optoelectronic memristor networks have excellent optical responsivity and the capability to execute the MAC operations, enabling its application in the real‐time processing of road vehicle images. Based on this, a convolutional kernel is constructed circuit for a single‐layer photoreceptor. The convolutional kernel is implemented via the 1 × 9 (M = 1, N = 9) STO:YSZ optoelectronic memristor network hardware. Upon inputting an image into the network, the eigenvalues of a specific edge are represented as the strength of the optical stimulation applied to the corresponding device in the network, and the network generates output (I) through MAC operations, as shown in **Figure** **6**a.

Figure 6b shows the edge detection procedure for images, using a 1000 × 600‐piexl road vehicle image as the input. The image is converted to grayscale, followed by feature extraction via a convolutional kernel. In the convolution operation on the image, a 3 × 3 kernel is used to slide over the input image with a stride of 1, decomposing the initial image into 81 sub‐images of 3 × 3. The pixel values of these images are translated into optical stimulation signals, sequentially fed into convolution kernels. Where the Sobel convolution kernel is implemented via a 2 × 9 (M = 2, N = 9) STO:YSZ optoelectronic memristor network, as shown in Figure 6d. During convolution, kernel weights map to device optical response values, with sub‐image‐convolution kernel dot product achieved via network MAC operations. The two Sobel convolution kernels are defined by the following equations:

(5) $$kernelx=\left[\begin{array}{ccc} 1 & \sqrt{2} & 1\\ 0 & 0 & 0\\ -1 & -\sqrt{2} & -1 \end{array}\right]$$

(6) $$kernely=\left[\begin{array}{ccc} -1 & 0 & 1\\ -\sqrt{2} & 0 & \sqrt{2}\\ -1 & 0 & 1 \end{array}\right]$$

Upon completion of convolution, the output images of kernels x and y are collected and merged into a single image. The formula for combining the two output images is defined in the following equation:

(7) $$I_{E}=I_{x}+I_{y}$$

where *I_x_* and *I_y_* correspond to the output current values of the convolution kernel x and y, respectively. Subsequently, the *I_E_* values were normalized to [0, 255]. Figure 6e‐Ι,II show the convolution outputs for the two kernels, with vehicle condition image features extracted in two orthogonal directions, respectively. The merged edge detection image of the real‐time vehicle condition, formed from the two normalized outputs, is shown in Figure 6e‐III, where the vehicle‐background edge is clearly visible. Additionally, the experimental result is highly consistent with the software‐derived vehicle edge information (Figure 6e‐IV), demonstrating the feasibility of edge detection based on STO:YSZ optoelectronic memristors.

Notably, crucial performance metrics are derived from the Figure 6e‐III edge detection results, as shown in Figure 6c. These metrics evaluate the experimental performance of the STO:YSZ memristor‐based edge detection algorithm against theoretical baselines, including PSNR, SSIM, F 1‐Score, and IoU. PSNR evaluates the pixel value difference of two images, where a larger value indicates smaller discrepancies. PSNR was calculated at 36.55 dB, indicating a negligible discrepancy between the detected image and the original, as corroborated by the output in Figure 6e. MSE reflects the discrepancy between predicted and true values, and the calculated value of 14.4011 indicates the edge detection model validity. Additionally, SSIM, F 1 Score, and IoU characterize image structural similarity, detected‐real edge consistency, and edge overlaps, respectively. Their calculated values are 0.8491, 0.9667, and 0.9356, all approach the theoretical maximum of 1. These results demonstrate the excellent performance of the STO:YSZ optoelectronic memristor‐based edge detection system, thereby establishing a new paradigm for practical applications in next‐generation real‐time information processing, machine vision, and autonomous driving.

## 3 Conclusion

In conclusion, this study demonstrates an optoelectronic memristor with a VAN structure of Pd/STO:YSZ/LSMO/STO/P‐Si. By the well‐aligned interfacial channels in STO:YSZ film, the device's ionic mobility is significantly enhanced, leading to high endurance and stable optical response. The device exhibits up to 10^8^ switching cycles of endurance and multi‐resistive states (retention time up to 10^4^ s). The device emulates diverse synaptic plasticity behaviors (PPF, LTP/LTD, and SADP, etc.), and achieves 91.5% accuracy in recognizing EMNIST letters via CNN. Meanwhile, it exhibits multi‐band optical response (405–650 nm), and stable resistive‐state dynamic switching with/without light using different power (10–100 mW) and wavelength laser sources. In a simple optoelectronic communication system, the device realizes optical image extraction and ASCII code optoelectronic signaling functions. Additionally, we implemented edge detection for road vehicle images using a convolutional kernel circuit constructed from memristor‐based network. These results demonstrate the potential of STO:YSZ optoelectronic memristors for high‐performance edge detection systems, with critical implications for real‐time tracking, autonomous driving, and intelligence transportation applications.

## 4 Experimental Section

### Device Fabrication

The STO:YSZ optoelectronic memristors analyzed in this work were fabricated using pulsed laser deposition (PLD) techniques. Heavily boron‐doped Si (001) was used as the substrate. The substrate was ultrasonically cleaned in deionized water for 5 min, followed by drying with pure N_2_. Before the film deposition, the chamber vacuum degree was maintained at 2×10^−4^ Pa. SrTiO_3_ and La_0_._67_Sr_0_._33_MnO_3_ films were deposited at 750 °C under 1 and 26 Pa atmospheres, respectively, with thicknesses of 48 and 35 nm. During the film's deposition, the distance between the sample and the target was maintained at 7 cm. SrTiO_3_:(Y_2_O_3_:ZrO_2_) (40 nm) was deposited on LSMO layers using a Physik COMPex Pro 20 S KrF excimer laser (λ= 248 nm) at 650 °C under 0.2 Pa O_2_ atmosphere, with a laser energy density of 1.3 J cm^−2^, a frequency of 5 Hz. Post‐deposition cooling to room temperature was conducted under 1000 Pa high‐oxygen pressure. Finally, the palladium electrodes, measuring 100 µm in diameter, were produced using a mask plate in an argon atmosphere at 1 Pa and a flow rate of 25 sccm through direct current magnetron sputtering.

### Characterization

AFM testing of the film was performed using a commercial MEP‐3D Origin microscope. The surface morphology of the device was characterized by a JEOL‐IT 800 Scanning Electron Microscope (SEM). The electrical performance tests were carried out with Keithley 2400, RIGOL DG 5102, RIGOL MSO4034. The optical responses were measured using laser sources at wavelengths of 405, 450, 520, and 650 nm with varying powers.

## Conflict of Interest

The authors declare no conflict of interest.

## Supporting information



Supporting Information

## Data Availability

The data that support the findings of this study are available in the supplementary material of this article.
