# Supplementary material for: High‐Endurance STO:YSZ Optoelectronic Memristors with Vertically Aligned Nanocomposite Structure for Edge Detection
Source: Adv Sci (Weinh). 2025 Nov 6;13(1):e13646. doi: 10.1002/advs.202513646 (PMC12767051; doi:10.1002/advs.202513646)
Supplement: Supplementary file 1 — Supporting Information [file ADVS-13-e13646-s001.docx]

**Supporting Information**

**High-Endurance STO:YSZ Optoelectronic Memristors with Vertically Aligned Nanocomposite Structure for Edge Detection**

*Jiacheng Wang*^1^, *Jikang Xu*^1^, *Xu Han*^1^, *Weidong Sun*, *Fu Wang*, *Zhen Zhao*, *Kangbo Zhao*, *Yufei Shang*, *Biao Yang*, *Hong Wang*, *Haoning Liu*, *Xiaobing Yan**

J. Wang, J. Xu, X. Han, W. Sun, F. Wang, Z. Zhao, K. Zhao, Y, Shang, B. Yang, H. Wang, H. Liu, X. Yan

Key Laboratory of Brain-Like Neuromorphic Devices and Systems of Hebei Province College of Electronic and Information Engineering, Hebei University

Baoding 071002, PR China

E-mail: [yanxiaobing@ime.ac.cn](mailto:yanxiaobing@ime.ac.cn) (X. Yan)

^1^ These authors contributed equally to the work


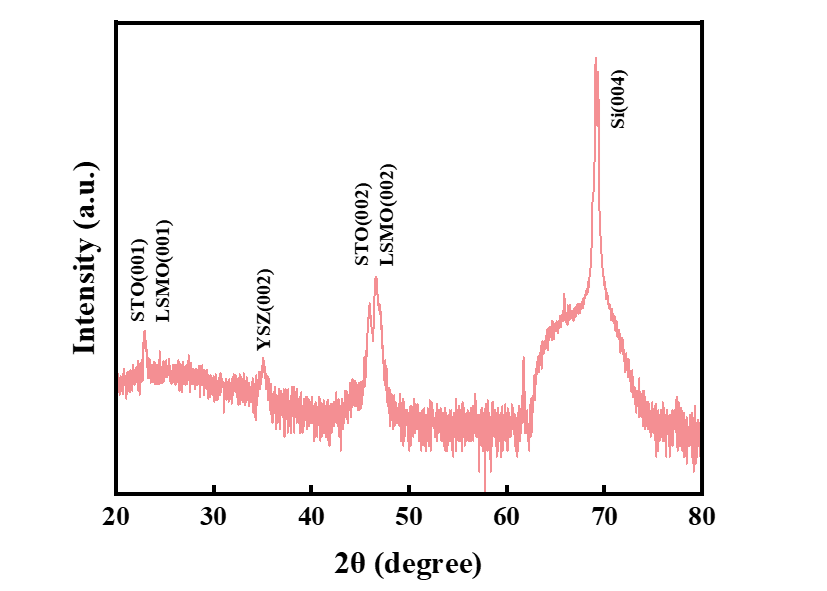


**Fig. S1.** XRD test results for STO:YSZ memristors.

**
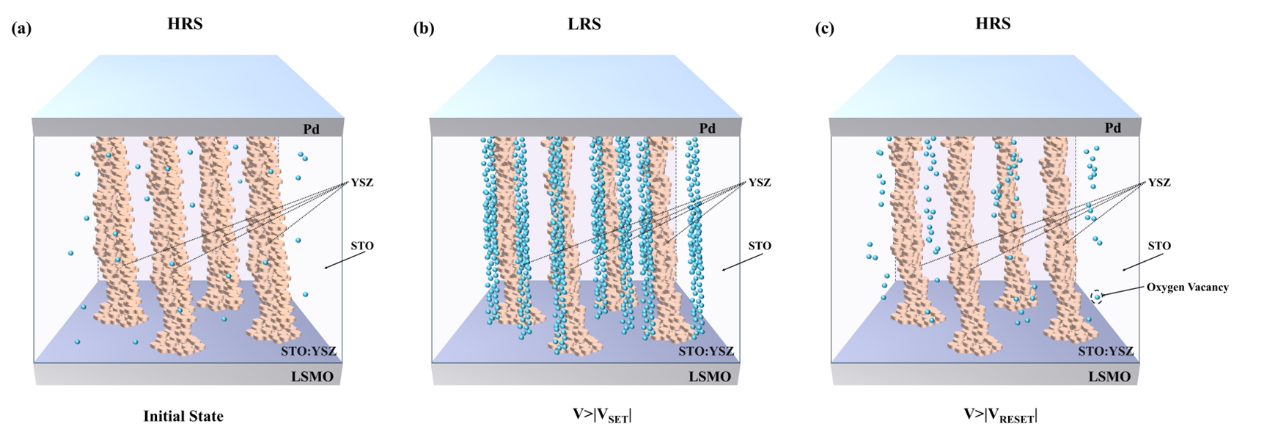
**

**Fig. S2.** Analysis results of the types and contents of elements in each thin film of the device cross−section low-resolution TEM testing.

The schematic diagram of the STO:YSZ memristors’ resistance switching process is shown in Figure S2. In VAN-structured films, oxygen vacancies readily form and accumulate at conductive channels in the perpendicular plane.^[1]^ Applying an external bias provides an effective region for oxygen vacancies to form conductive channels.^[2]^ As shown in Figure S2a, when no voltage bias is applied, the device remains in its initial state (HRS), with a low number of oxygen vacancies sparsely distributed within the STO:YSZ functional layer. When the top electrode (Pd) is applied with a positive voltage, the number of oxygen vacancies within the film begins to gradually increase. Under the influence of the electric field, oxygen vacancies gradually merge and migrate toward the Pd. When positive voltage exceeds V_SET_ (V>|V_SET_|), oxygen vacancies aggregate to form oxygen vacancy channels connecting Pd and LSMO along the vertical conductive pathway, causing the device to transition from HRS to LRS, as shown in Figure S 2b. Conversely, under reverse bias, the oxygen vacancies channels present in the vertical conductive pathways start to disperse. When the voltage exceeds V_RESET_ (V>|V_RESET_|), the oxygen vacancy channel breaks, causing the device to switch from LRS to HRS, as shown in Figure S 2c. This is consistent with similar reports.^[3, 4]^


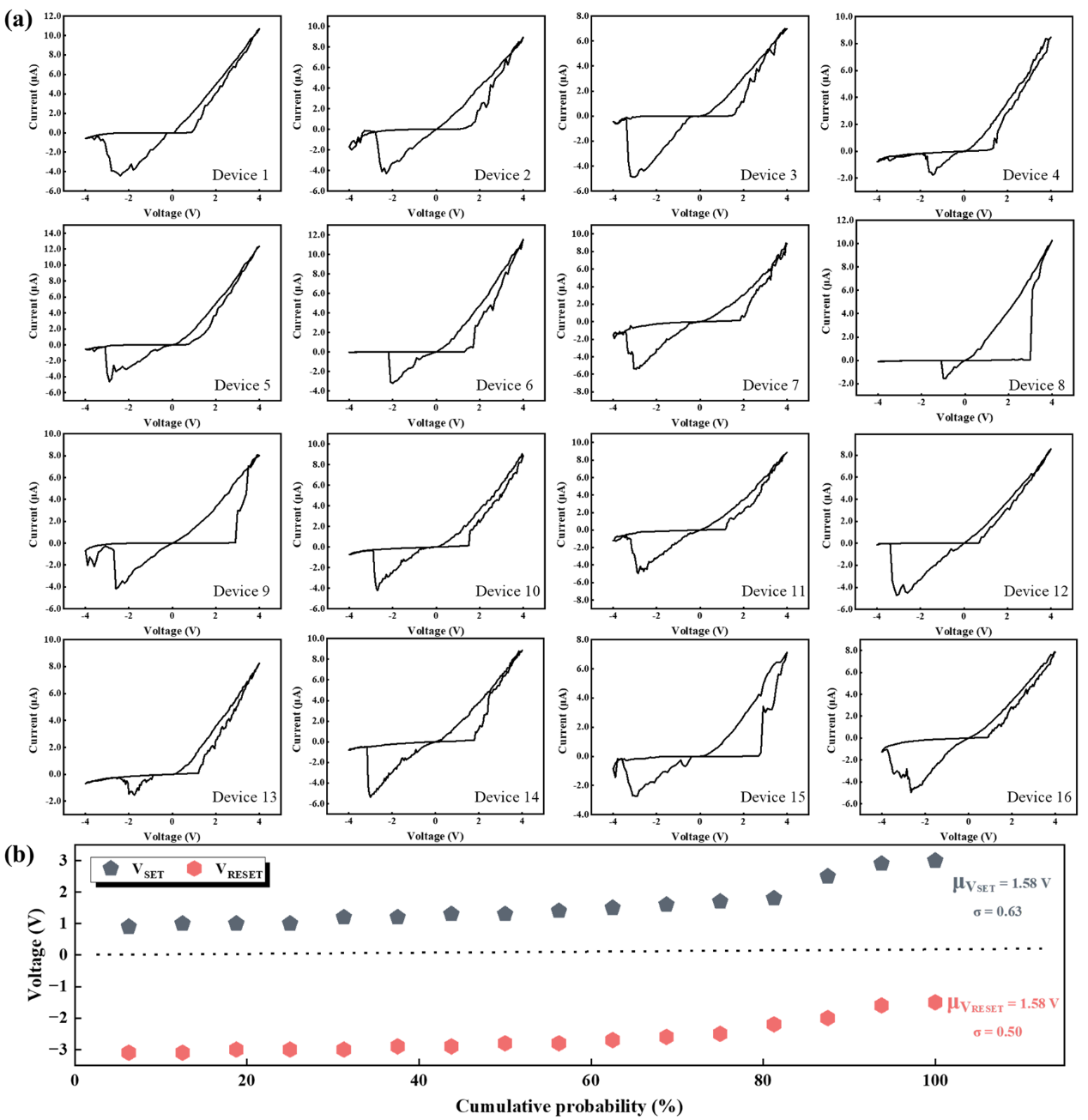


**Fig. S3.** (a) *IV* curves of 16 randomly selected devices. (b) Cumulative Probability distribution of SET/RESET voltage of 16 selected STO:YSZ memristors.


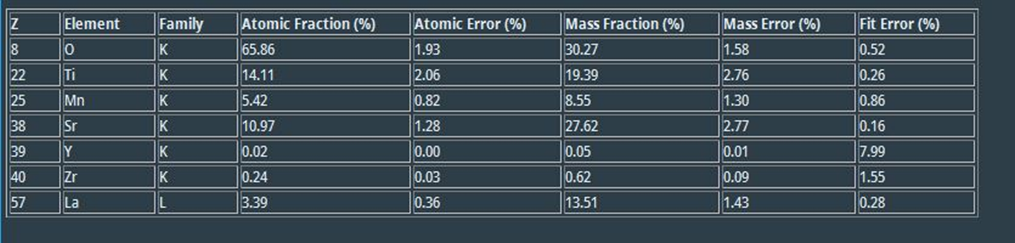


**Fig. S4.** Analysis results of the types and contents of elements in each thin film of the device cross−section low-resolution TEM testing.


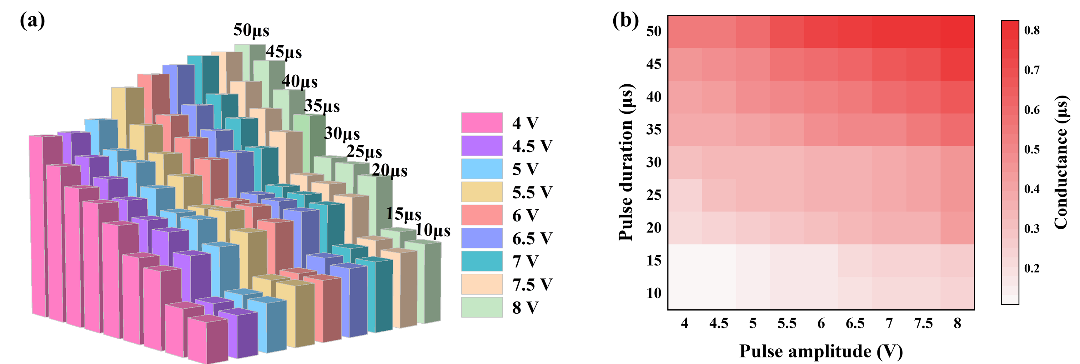


**Fig. S5.** (a) The Influence of Pulse Amplitude and Duration on the Conductivity of $\text{9×}\text{9}$ STO:YSZ Memristor Array. (b) Thermal map of conductivity value statistics for $\text{9×}\text{9}$ device array.


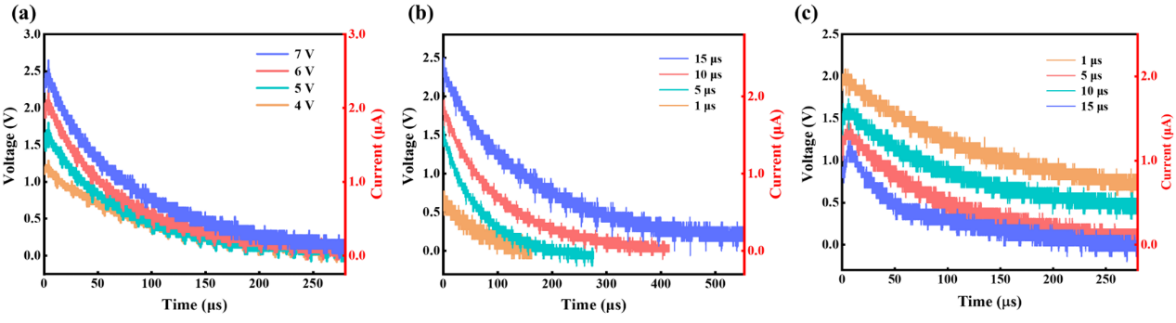


**Fig. S6.** (a) Changes in EPSC of STO:YSZ memristor under different amplitude pulse sequences. (b) Changes in EPSC of STO:YSZ Memristor under Different Duration Pulse Sequence. (c) Changes in EPSC of STO:YSZ memristor under different time interval sequences.


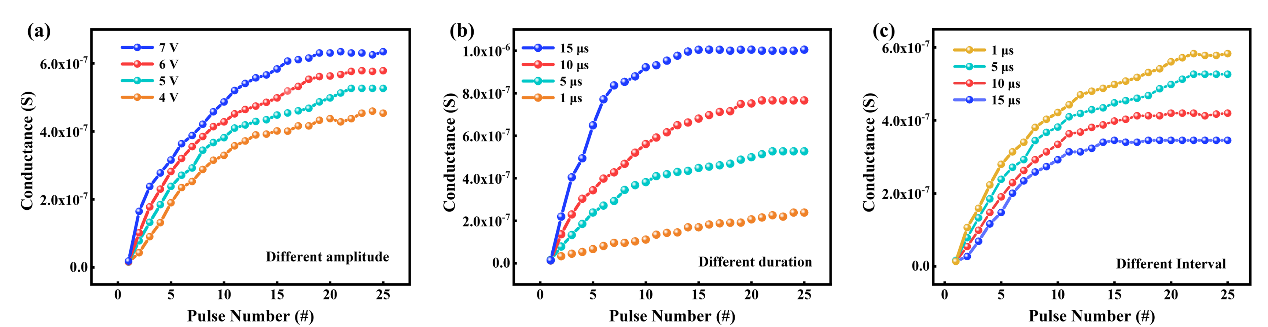


**Fig. S7.** (a) The influence of different amplitudes in 30 cycles on the conductance modulation of a single device, with a pulse width of 5 μs and an interval of 5 μs. (b) The influence of different durations of 30 cycles on the conductance modulation of a single device, with a pulse width of 5 μs and an interval of 5 μs. (c) The influence of different time intervals of 30 cycles on the conductivity modulation of a single device, with a pulse width of 5 μs and an interval of 5 μs.


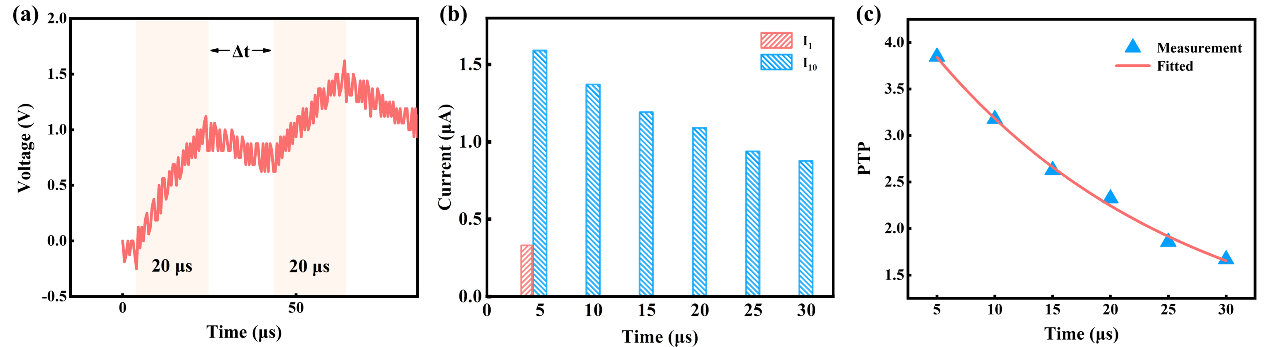


**Fig. S8.** (a) Changes in current response of STO:YSZ after pulse application. (b) Current-response statistics of STO:YSZ devices under the tenth pulse stimulation (with time intervals of 5 μs, 10 μs, 15 μs, 20 μs, 25 μs, and 30 μs). (c) The index statistics of PTP.

As shown in Fig. S5(c), the PTP index can be obtained from equation:

$\text{PTP=}{(I_{10}-I_{1})}/{I_{1}}\text{×100\%}$ (1)

where *I_10_* denotes the maximum current response of the STO:YSZ memristor under the 10th pulse stimulation. The fitting formula for the PTP index of the STO:YSZ memristor is identical to that of PPF.


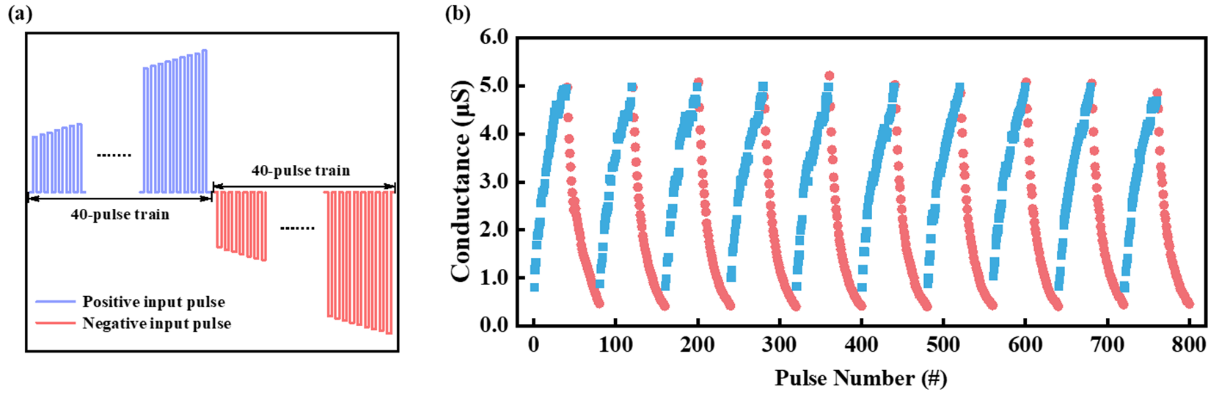


**Fig. S9.** (a) Schematic diagram of pulse testing for linear LTP/LTD. (b) 10 sets of LTP and LTD repeatability tests for the device。


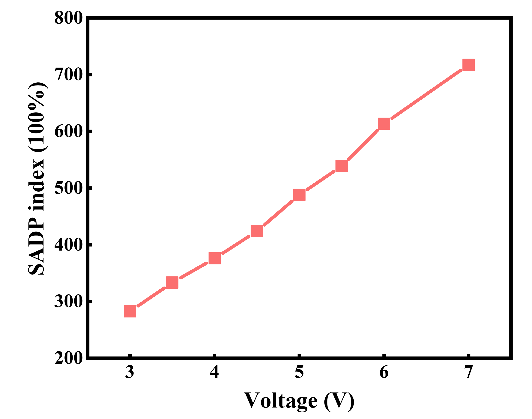


**Fig. S10.** SADP index statistics($\text{I}_{\text{25}}\text{/}\text{I}_{\text{1}}\text{×100\%}$).


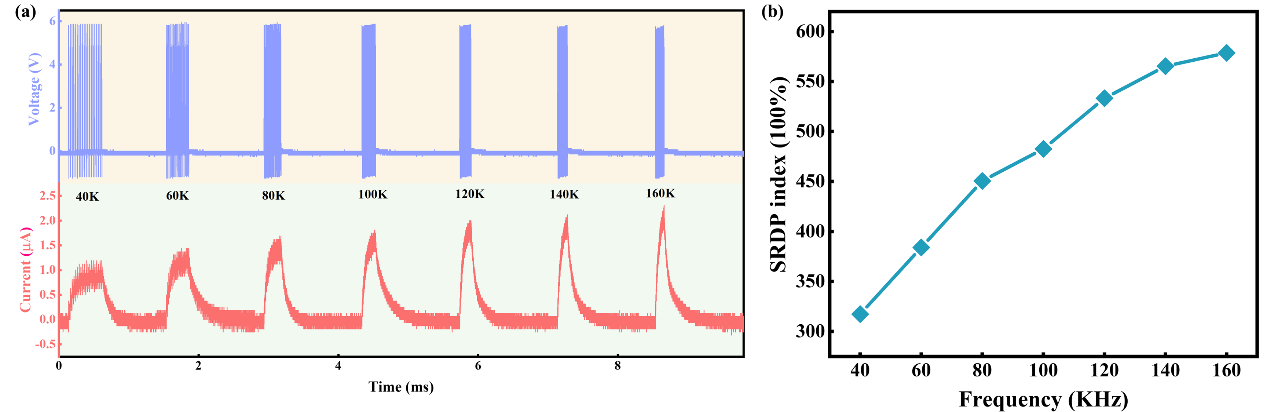


**Fig. S11.** (a) Simulation of SRDP learning mechanism using STO:YSZ memristor, with a pulse number N of 20, a fixed amplitude of 5V, a duration of 5 μs, and varying frequencies of 40 kHz, 60 kHz, 80 kHz, 100 kHz, 120 kHz, 140 kHz, and 160 kHz. (b) SRDP index statistics($\text{I}_{\text{20}}\text{/}\text{I}_{\text{1}}\text{×100\%}$).


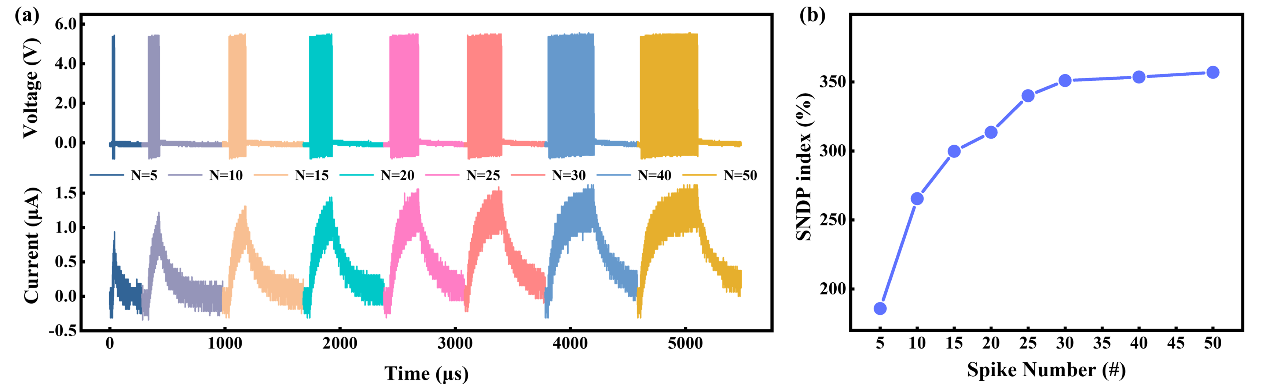


**Fig. S12.** (a) Simulation of SNDP learning mechanism using STO:YSZ memristor, Fixed pulse amplitude, width, and interval remain unchanged, and the number of applied pulses N is changed to 5, 10, 15, 20, 25, 30, 40, and 50. (b) SNDP index statistics($\text{I}_{\text{N}}\text{/}\text{I}_{\text{1}}\text{×100\%}$).


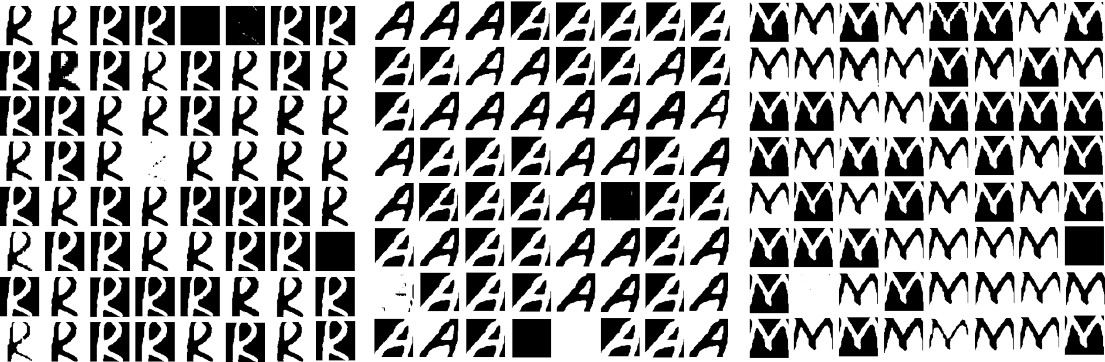


**Fig. S13.** Visualization presentation of feature maps for each channel after the first layer of convolution, generating 64 feature images for each letter.


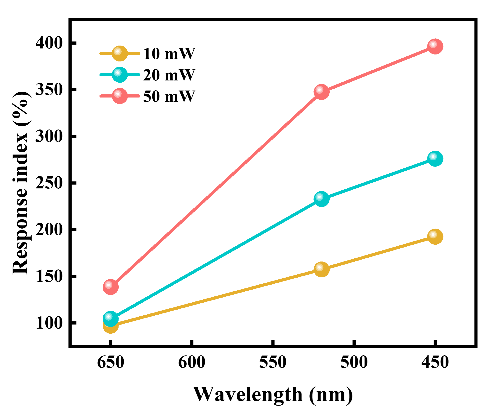


**Fig. S14.** Response index of STO:YSZ memristor to light stimuli of different wavelengths and powers.


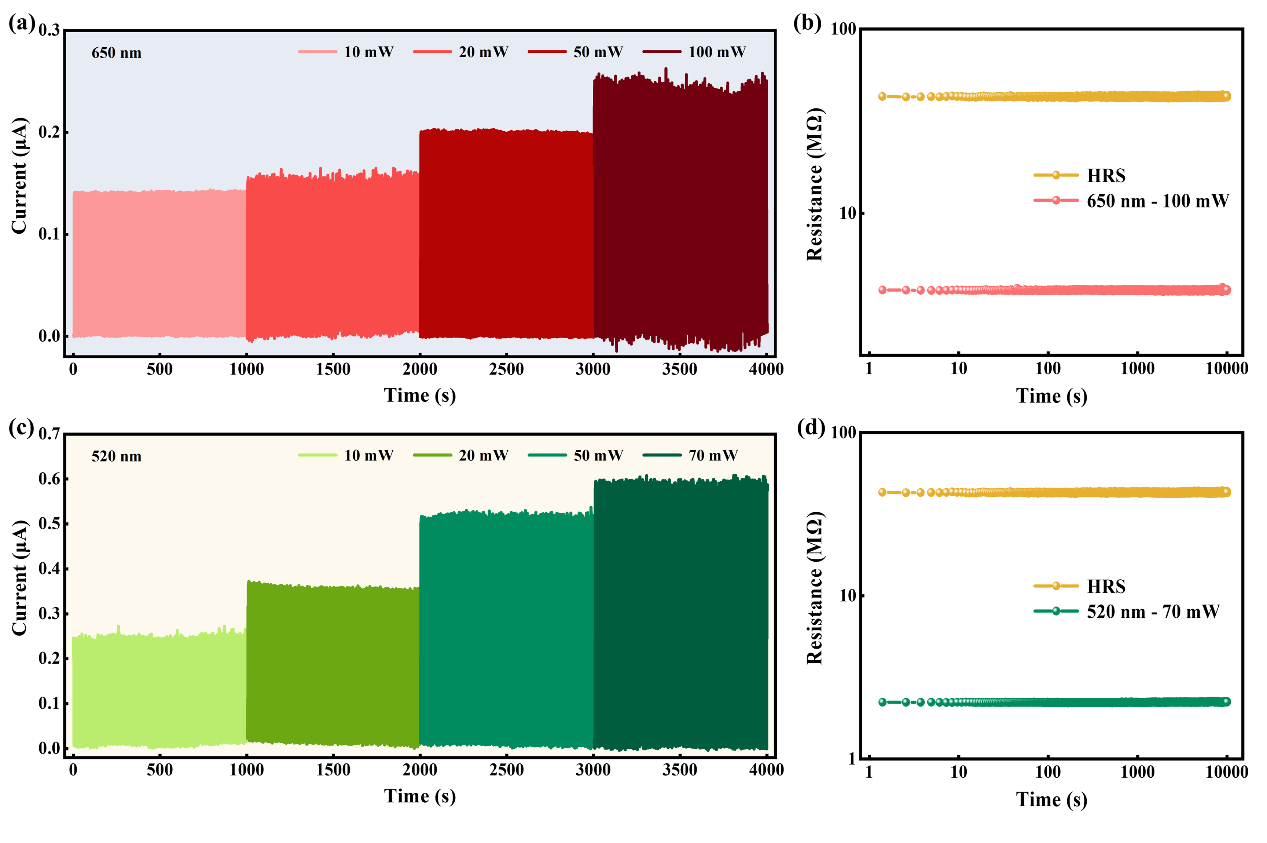


**Fig. S15.** (a) The stable characteristics of Pd/STO:YSZ/LSMO/STO/P-Si memristors under light stimulation at a wavelength of 650 nm were measured by 100 cycles of optical switching at powers of 10 mW, 20 m, 50 mW, and 100 mW, respectively. (b) STO:YSZ memristor retention characteristics test under continuous light stimulation at a wavelength of 650 nm and a power of 100 mW. (c) The stable characteristics of Pd/STO: YSZ/LSMO/STO/P-Si memristors under light stimulation at a wavelength of 520 nm were measured by 100 cycles of optical switching at powers of 10 mW, 20 m, 50 mW, and 70 mW, respectively. (d) STO:YSZ memristor retention characteristics test under continuous light stimulation at a wavelength of 520 nm and a power of 70 mW.


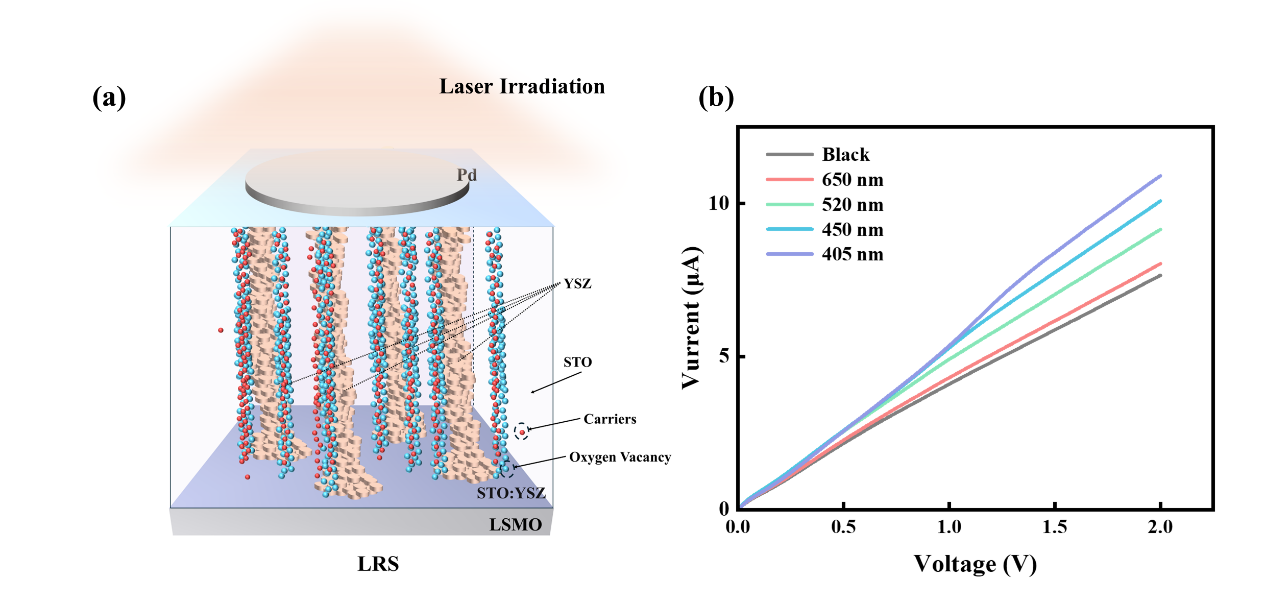


**Fig. S16.** (a) Distribution of photogenerated carriers within the STO:YSZ film under illumination. (b) Current response of STO:YSZ devices under illumination of different wavelengths.

Figure S15a shows the distribution of carriers in the STO:YSZ film under illumination when the device is in the LRS. In the STO:YSZ thin film with VAN structure, both components tend to grow along the direction with lower surface energy,^[5]^ oxygen vacancies are likely to be generated and accumulated at the interface between the two components (as shown by the blue spheres in Figure S a).^[1]^ High - density conductive channels can promote the rapid migration of oxygen ions and become effective regions for the formation of oxygen vacancy channels.^[2]^ As shown in Figure S2 a, when no voltage bias is applied, the device is in the initial state (HRS). The number of oxygen vacancies in the STO:YSZ thin film is relatively small and randomly distributed. After applying light illumination, the number of photogenerated carriers increases and are effectively captured by the oxygen vacancy channels (as shown by the distribution of the red spheres in Figure S a). Oxygen vacancies, acting as capture centers for carriers,^[6]^ can significantly improve the lifetime of photogenerated carriers.^[7]^ Through the VAN structure, photogenerated carriers can rapidly migrate along perpendicular conductive pathways. Additionally, we investigated the photocurrent response of the device under illumination at different wavelengths, as shown in Figure S15b. Set the device’s resistance state to LRS, configure the DC scan range at 0-2 V, and vary the light wavelength (405-650 nm). As the wavelength decreases, the LRS current exhibits an increasing trend. This negative correlation with wavelength is consistent with the test results shown in Figure 4a. Compared to dark current, the response of the device shows a significant improvement when illuminated. This can be attributed to the formation of oxygen vacancy channels within the conductive pathways, which effectively enhance the migration efficiency of photogenerated carriers.


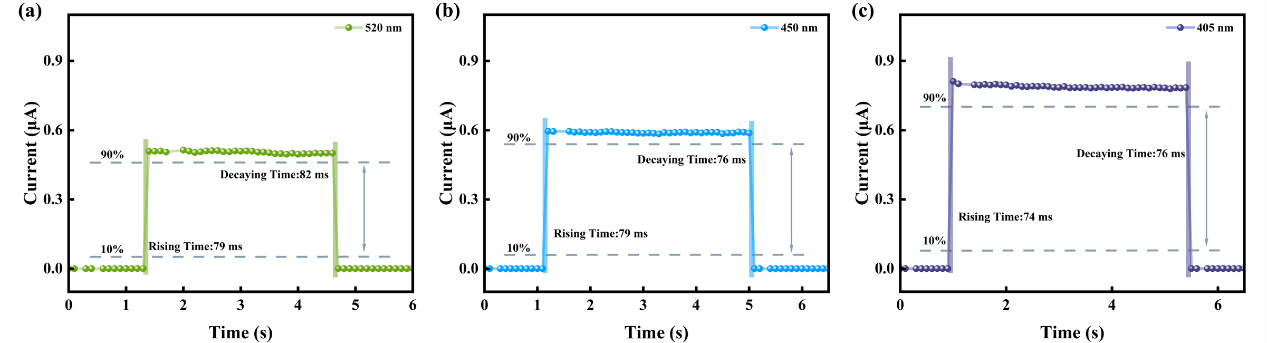


**Fig. S17.** (a) Response time test of STO:YSZ memristor under light stimulation with a wavelength of 520 nm and a power of 50 mW, where the rise time is 79 ms and the decay time is 82 ms. (b) Response time test of STO:YSZ memristor under light stimulation with a wavelength of 450 nm and a power of 50 mW, where the rise time is 79 ms and the decay time is 76 ms. (c) Response time test of STO:YSZ memristor under light stimulation with a wavelength of 405 nm and a power of 50 mW, where the rise time is 74 ms and the decay time is 76 ms. STO:YSZ optoelectronic devices have stable light response speed (in the millisecond range) and almost identical response speed to different wavelengths of light.


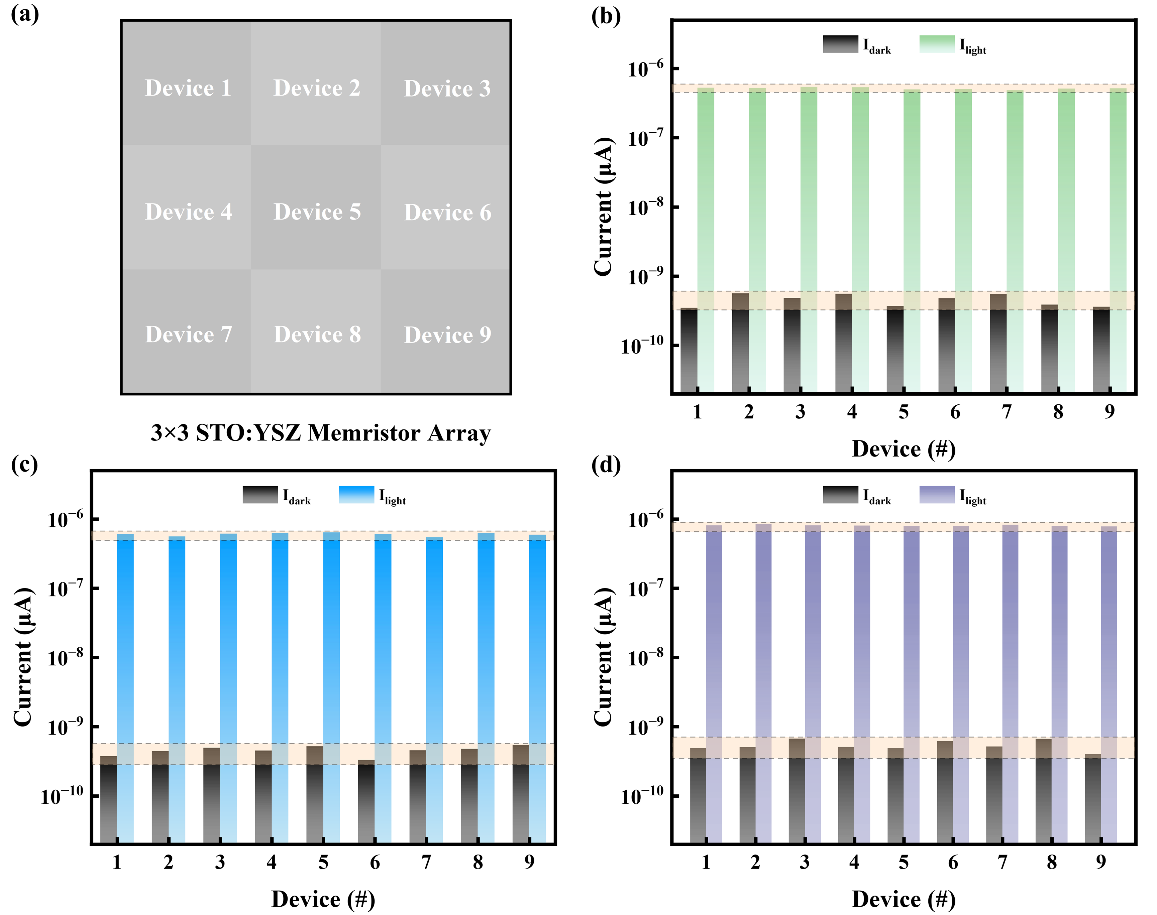


**Fig. S18.** (a) $\text{3}\text{×}\text{3}$ STO:YSZ Optoelectronic Memristor Array and Device Number. (b) Statistical analysis of light dark current response of 9 STO:YSZ photoresistors in the array under light stimulation with/without a wavelength of 520 nm and a power of 50 mW. (c) Statistical analysis of light dark current response of 9 STO:YSZ photoresistors in the array under light stimulation with/without a wavelength of 450 nm and a power of 50 mW. (d) Statistical analysis of light dark current response of 9 STO:YSZ photoresistors in the array under light stimulation with/without a wavelength of 405 nm and a power of 50 mW. Among them, the read voltage is set to 1 V. The light dark current response of these 9 devices shows good consistency, avoiding the influence of randomness.

**Reference**

[1] H. Wang, M. Cui, G. Fu, J. Zhang, X. Ding, I. Azaceta, M. Bugnet, D.M. Kepaptsoglou, V.K. Lazarov, V.A. de la Peña O’Shea, F.E. Oropeza, K.H.L. Zhang, Vertically aligned Ni/NiO nanocomposites with abundant oxygen deficient hetero-interfaces for enhanced overall water splitting, Science China Chemistry 65(10) (2022) 1885-1894. <https://doi.org/10.1007/s11426-022-1326-2>.

[2] K. Li, J. Zhang, Y. Chen, J. Pan, Y. Zheng, S. Xu, R. Zhao, M. Li, R. Qi, R. Huang, Z. Yan, P. Yu, J.M. Liu, J.L. MacManus-Driscoll, H. Yang, W. Li, In situ observation of oxygen ion dynamics in topological phase change memristors through self-assembled interface design, Sci Adv 11(33) (2025) eadw8513. <https://doi.org/10.1126/sciadv.adw8513>.

[3] J. Xu, Y. Zhang, J. Wang, W. Zhang, T. Li, K. Zhang, X. Yan, Ultra-Low Power and Robust Bi(2)SeO(5) Films Optoelectronic Memristors for Bio-Visual Perception Computing Systems, Adv Mater (2025) e2509174. <https://doi.org/10.1002/adma.202509174>.

[4] X. Ren, Y. Nan, X. Chen, R. Ding, H. Zheng, Research on Resistive Switching and Synaptic Performance of CeOx/TaOx-Based Memristor, ACS Applied Electronic Materials 7(11) (2025) 5030-5040. <https://doi.org/10.1021/acsaelm.5c00483>.

[5] J.L. MacManus‐Driscoll, Self‐Assembled Heteroepitaxial Oxide Nanocomposite Thin Film Structures: Designing Interface‐Induced Functionality in Electronic Materials, Advanced Functional Materials 20(13) (2010) 2035-2045. <https://doi.org/10.1002/adfm.201000373>.

[6] D. Zu, Y. Ying, Q. Wei, P. Xiong, M.S. Ahmed, Z. Lin, M.M. Li, M. Li, Z. Xu, G. Chen, L. Bai, S. She, Y.H. Tsang, H. Huang, Oxygen Vacancies Trigger Rapid Charge Transport Channels at the Engineered Interface of S-Scheme Heterojunction for Boosting Photocatalytic Performance, Angew Chem Int Ed Engl 63(31) (2024) e202405756. <https://doi.org/10.1002/anie.202405756>.

[7] X. Bai, K. Luo, W. Cui, Z. Wang, Z. Ma, X. Wang, W. Zhang, X. Cui, Oxygen vacancy induced carrier localization in enhancing photocatalytic performance of ZnO, Vacuum 221 (2024). <https://doi.org/10.1016/j.vacuum.2023.112940>.
